# A Rare Case of Mesalazine-Induced Acute Myocarditis in a 19-Year-Old Female With Ulcerative Colitis

**DOI:** 10.7759/cureus.20036

**Published:** 2021-11-30

**Authors:** Abuobeida Ali, Aravind Sunderavel Kumaravel Kanagavelu, Abdulhameed Rahimi, Zia Mehmood, Adeel B Tariq, Tapas Das, Ali Elmdaah

**Affiliations:** 1 Gastroenterology, Peterborough City Hospital, Peterborough, GBR; 2 Internal Medicine, Peterborough City Hospital, Peterborough, GBR; 3 Cardiology, Peterborough City Hospital, Peterborough, GBR

**Keywords:** cardiac magnetic resonance imaging, inflammatory bowel disease, cardiac mri, myocarditis, mesalazine, ulcerative colitis

## Abstract

Mesalazine is a commonly used first-line therapy to treat acute mild to moderate ulcerative colitis (UC). Myocarditis is a rare complication of inflammatory bowel disease. This is a case report of a 19-year-old female with myocarditis induced after commencing mesalazine for UC. She was admitted with pleuritic-type chest pain associated with severe dyspnoea and was hemodynamically unstable during admission. She had elevated troponin and N-terminal pro-B-type natriuretic peptide. Transthoracic echocardiogram (TTE) results suggested there was evidence of myocarditis with reduced ejection fraction, which was later confirmed by cardiac magnetic resonance imaging. There was a rapid improvement of cardiac status after stopping mesalazine within two weeks.

## Introduction

Inflammatory bowel disease (IBD) is a disorder causing chronic inflammation of the gastrointestinal tract. IBD types include ulcerative colitis (UC) and Crohn’s disease (CD). Mucosal healing (Mayo endoscopic scores (MES) 0) remains the mainstay of treatment for IBD. Mesalazine is a commonly used first-line therapy to treat acute attacks of mild to moderate ulcerative colitis (UC) and maintenance of remission [1].

Myocarditis is a rare complication of IBD with an incidence of 0.3% [2]. But mesalazine-induced myocarditis is an even rarer but recognized complication and may develop potentially to cause cardiogenic shock and death [3]. We present a case of a 19-year-old female with myocarditis induced after starting mesalazine for UC and conduct a review of the literature.

## Case presentation

A 19-year-old, otherwise fit-and-well female presented to our hospital with an acute flare-up of UC with bloody diarrhea for a few days. She was a non-smoker and non-alcoholic. Based on the results of flexible sigmoidoscopy and biopsy, the diagnosis of moderate UC was established and a maximum dose of oral mesalazine 2.4 grams a day and a tapering dose of oral prednisolone (starting at 30 mg OD) were prescribed. She responded well to treatment, reporting improved abdominal symptoms.

However, four weeks later, she was readmitted with a central, sharp, pleuritic-type chest pain associated with severe dyspnoea without any prodromal symptoms like fever, myalgia, or cough. On initial assessment in the emergency department, she was dyspnoeic, tachycardic with a blood pressure of 80/60 mmHg and oxygen saturation of 80% requiring 10 liters of oxygen to be maintained above 94%. The patient’s cardiovascular and respiratory examinations were unremarkable.

Her resting 12-lead electrocardiogram (ECG) revealed sinus tachycardia without any ST changes. There was an elevated cardiac troponin-T enzyme of 64 ng/ml (laboratory normal of <5 ng/mL) and normal serum electrolytes. Based on this, she was treated for acute coronary syndrome with dual antiplatelet therapy and was admitted to the coronary care unit (CCU). Meanwhile, she was also investigated to rule out pulmonary embolism. D-dimer was negative, and computed tomography of pulmonary angiogram was unremarkable.

In the next few days, our patient further developed features of heart failure and cardiogenic shock for which she was treated with inotropes and furosemide infusion to offload. N-terminal pro-B-type natriuretic peptide (NT-Pro BNP) came back as >34,000 pg/mL (laboratory normal of <300 pg/mL), and chest X-ray confirmed the evidence of pulmonary edema. Transthoracic echocardiogram (TTE) results suggested that there is evidence of dilated cardiomyopathy and myocarditis with severely impaired left ventricular systolic function and ejection fraction (LVEF) of 31%. Following this, cardiac magnetic resonance imaging (CMR) was performed, which confirmed myocarditis. The viral, autoimmune, metabolic, and vasculitic screens came back negative.

Based on the above investigations, the diagnosis of acute myocarditis was made after excluding other aetiologies. Subsequently, mesalazine was stopped immediately and analgesics were prescribed to alleviate pain. She was then referred to the gastroenterology team regarding further advice on managing ulcerative colitis for which they suggested biologic therapy with “infliximab” as per local trust guidelines. The rapid improvement in the patient's cardiac status after drug discontinuation supported the diagnosis of myocarditis induced by mesalazine.

Repeat TTE in two weeks' time showed an improvement in left ventricular ejection fraction (LVEF) of 48%. The two-month follow-up TTE showed complete recovery with a 59% LVEF with normal biventricular size and function (Table 1). There was reduced gadolinium enhancement of the epicardium and myocardium in the follow-up CMR confirming recovery. Recent TTE done two years later showed LVEF of 61% confirming there is no residual myocarditis (Table 1).

**Table 1:** Timeline and cardiac investigations LVEF: left ventricular ejection fraction; NT-Pro BNP: N-terminal pro-b-type natriuretic peptide

	At the time of presentation	2 months after stopping mesalazine	1 year after stopping mesalazine
LVEF %	31%	59%	61%
Troponin ng/L	63	<5	<5
NT-Pro BNP pg/mL	>34,000	871	<300

## Discussion

Mesalazine is a form of aminosalicylate (5-ASA) that does not contain a sulfa group. Mesalazine (5-ASA) is a commonly used IBD drug because of its anti-inflammatory action on colonic epithelial cells [4]. Though this mechanism is still unclear, research shows that mesalazine produces its effects by inhibiting pro-inflammatory mediators like Roms, Leukotrienes, Interleukin 1, and Tumour Necrosis Factor-alpha (TNF α) [4]. Like any drug, mesalazine also has side effects but myocarditis is one of the rare or very rare complications of mesalazine [3].

Myocarditis simply means the inflammation of the heart muscles. In the acute type of myocarditis, patients usually present with chest pain, dyspnoea, or palpitations [5]. These symptoms are common for most cardiovascular symptoms and hence further investigation is necessary to exclude other causes. Laboratory investigations show raised troponin levels. ECG might reveal non-specific changes like sinus tachycardia, ST-wave, and T-wave abnormalities. Echocardiograms might show new wall motion abnormalities. Among the non-invasive investigations, cardiac MRI is ideal for confirming the diagnosis [5-6]. The criteria for suspecting myocarditis includes cardiovascular symptoms alone with either raised cardiac enzymes or ECG changes or abnormal imaging (echo or cardiac MRI). However, immunohistology remains the gold-standard investigation for diagnosing acute myocarditis.

The common causes of myocarditis include bacterial (like Staphylococcus, Streptococcus, etc.), viral (Coxsackieviruses, adenoviruses, etc.), fungal (Aspergillus, Actinomyces, etc.), and parasitic (Trichinella, Echinococcus, etc.) infections. Chemicals, drug therapies, and autoimmune diseases can also cause acute myocarditis. But it is also well-established that patients with inflammatory bowel diseases are at a higher risk of developing myopericarditis [5,7]. But there is no particular symptom, investigations, imaging studies, or immune-histological evidence that are unique to mesalazine-induced myocarditis and differentiate it from the others.

Based on an extensive literature search, we can note that the onset of symptoms from starting mesalazine has mostly been around two to four weeks time, which was similar to our patient’s presentation. As per our literature review, all nine patients had a raised troponin level, which was again similar to our case. Most cases either showed sinus tachycardia or nonspecific ST changes. The majority of the cases from our literature documents showed myocarditis changes with reduced ejection fraction, in some cases up to 25% on TTE and CMR (Table 2).

**Table 2:** A summary of case studies about mesalazine-induced myocarditis EF: ejection fraction

Author (Reference)	Age, gender	Type of inflammatory bowel disease	Duration of mesalazine given	Troponin	12-lead electrocardiogram	Transthoracic echocardiogram (Ejection fraction)	Cardiac magnetic resonance imaging	Treatment & Outcome
Carlos Galvao Braga [8]	19 years, male	Crohn’s disease	2 weeks	Peak troponin I: 27.3 ng/ml	Slight ST-segment elevation with an upward concavity in leads I, II, III, aVF, and V3–V6.	Global hypocontractility (EF: 38%)	Multiple areas of myocardial fibrosis, mainly sub-epicardium.	Cessation of mesalazine & improved well in one week.
Amira Ibrahim [9]	21 years, male	Crohn’s disease	4 weeks	Troponin I: 2.21 ng/ml	First-degree heart block and non-specific ST-T changes.	Normal wall motion (EF: 55-60%)	Subepicardial to mid-myocardial delayed gadolinium hyper-enhancement and edema involving the basal inferior to the inferolateral wall.	Cessation of mesalazine & marked improvement in 48 hours.
William L Baker [10]	38 years, male	Crohn’s disease	3 weeks	Troponin I: 3.37 ng/ml	Sinus tachycardia with T-wave flattening.	Mildly impaired left ventricular systolic function and inferolateral wall hypokinesia (EF:40%)	Mild global hypokinesis of the left ventricle, and patchy mid-myocardial late gadolinium enhancement in the posterior wall of the left ventricle and, to a lesser degree, in the inferior interventricular septum.	Cessation of mesalazine, metoprolol succinate & resolution of symptoms in 4 days.
Kelechukwu U. Okoro [11]	23 years, male	Ulcerative colitis	6 months	Troponin 14.55 ng/dl	Sinus tachycardia without ischaemic changes.	Akinetic apex (EF: Preserved)	Linear delayed hyper-enhancement involving the mid-myocardium of the distal septum.	Cessation of mesalazine, methylprednisolone started & the condition improved.
Mohamed E. Taha [12]	18 years, female	Ulcerative colitis	2 weeks	Peak troponin I 5.59 µg/L	Sinus tachycardia, borderline T-wave abnormalities in leads II, III and AVF	Normal left ventricular size, thickness, systolic and diastolic function. (EF:55%)	Delayed enhancement showed trace pericardial effusion.	Cessation of mesalazine & symptoms resolved in 48 hours.
Shiva T. Radhakrishnan [13]	49 years, male	Ulcerative colitis	2 weeks	Troponin T 146 ng/ml	Sinus tachycardia	Not mentioned in the article.	Subepicardial delayed gadolinium enhancement in the basal to middle inferior and inferolateral segments of the heart with matching high signal intensity seen on T2-weighted images of the same area.	Cessation of mesalazine & improvement in patient’s condition in 3 days.
Michele Sorleto [14]	18 year, male	Crohn’s disease	6 months	Troponin hs:1158 pg/ml	ST-segment elevation with an upward concavity in leads II, III, aVF, and V4–V6	Not mentioned in the article.	Late gadolinium enhancement and myopericardial edema.	Cessation of mesalazine and symptoms normalized within 7 days.
Thomas Kingston [15]	23 years, male	Ulcerative colitis	5 months	Troponin 14.5 ng/ml	Not mentioned in the article.	Akinetic apex.	Linear delayed hyper-enhancement involving the mid-myocardium of the distal septum.	Cessation of mesalazine, commencement of IV steroids & prompt resolution of symptoms
Thomas Mellor [16]	27 years, male	Crohn’s disease	3 weeks	Troponin I: 0.92 ng/ml	No ischaemic changes.	Biventricular dilation, (EF:25-30%)	Late enhancement of the subepicardial lateral wall.	Cessation of mesalazine & improvement of symptoms.

The definitive treatment of mesalazine-induced myocarditis has been the abrupt cessation of the drug. In most patients, it yielded a rapid resolution of symptoms without any chronic residual changes. In a few patients, short courses of steroids have been used although it’s still not clear and indicated. Our patient improved drastically after discontinuing medication without the use of steroids. Her follow-up scan in three years’ time reassured that there were no chronic changes because of the toxicity (Table 2).

## Conclusions

This patient’s case illustrates that clinicians should be more alert and aware of this very rare but potentially life-threatening adverse effect of mesalazine because this drug is widely prescribed for patients with IBD. It is essential to consider myocarditis as an important differential diagnosis of chest pain in a patient taking mesalazine because the primary management simply involves discontinuing the medication. This would improve the disease quite rapidly without causing permanent after-effects.

## References

[1] Lamb CA, Kennedy NA, Raine T (2019). British Society of Gastroenterology consensus guidelines on the management of inflammatory bowel disease in adults. Gut.

[2] Zakko SF, Gordon GL, Murthy U (2016). Once-daily mesalamine granules for maintaining remission of ulcerative colitis: pooled analysis of efficacy, safety, and prognostic factors. Postgrad Med.

[3] Brown G (2016). 5-aminosalicylic acid-associated myocarditis and pericarditis: a narrative review. Can J Hosp Pharm.

[4] Caprilli R, Cesarini M, Angelucci E, Frieri G (2009). The long journey of salicylates in ulcerative colitis: the past and the future. J Crohns Colitis.

[5] Caforio AL, Pankuweit S, Arbustini E (2013). Current state of knowledge on aetiology, diagnosis, management, and therapy of myocarditis: a position statement of the European Society of Cardiology Working Group on Myocardial and Pericardial Diseases. Eur Heart J.

[6] Dominguez F, Kühl U, Pieske B, Garcia-Pavia P, Tschöpe C (2016). Update on myocarditis and inflammatory cardiomyopathy: reemergence of endomyocardial biopsy. Rev Esp Cardiol (Engl Ed).

[7] Mitchell NE, Harrison N, Junga Z, Singla M (2018). Heart under attack: cardiac manifestations of inflammatory bowel disease. Inflamm Bowel Dis.

[8] Braga CG, Martins J, Arantes C (2013). Mesalamine-induced myocarditis following diagnosis of Crohn's disease: a case report. Rev Port Cardiol (English Edition).

[9] Ibrahim A, Khalil C, Megaly M, Tawadros M, Mosleh W, Corbelli J (2019). Mesalamine-induced myocarditis in a young athlete: can he run again?. Cureus.

[10] Baker WL, Saulsberry WJ, Elliott K, Parker MW (2015). Cardiac MRI-confirmed mesalamine-induced myocarditis. BMJ Case Rep.

[11] Okoro KU, Roby MD, Bankole AA (2018). Myocarditis secondary to Mesalamine-Induced cardiotoxicity in a patient with ulcerative colitis. Case Rep Med.

[12] Taha ME, Abdalla A, Al-Khafaji J, Malik S (2019). Mesalamine-induced myopericarditis: a case report and literature review. Cardiol Res.

[13] Radhakrishnan ST, Mohanaruban A, Hoque S (2018). Mesalazine-induced myocarditis: a case report. J Med Case Rep.

[14] Sorleto M, Dürrwald S, Wiemer M (2015). Mesalazine-induced myopericarditis in a patient with a recent diagnosis of Crohn’s disease: apropos of a case. Case Rep Cardiol.

[15] Kingston T, Adham K, Harb R (2016). Mesalamine Inducing Myocarditis: An Atypical Complication. Am J Gastroenterol.

[16] Mellor T, Junga Z, Aduli Aduli (2012). A rare complication of 5-ASA therapy. Am J Gastroenterol.

